# Electrosprayed microparticles for intestinal delivery of prednisolone

**DOI:** 10.1098/rsif.2018.0491

**Published:** 2018-08-29

**Authors:** T. Shams, U. E. Illangakoon, M. Parhizkar, A. H. Harker, S. Edirisinghe, M. Orlu, M. Edirisinghe

**Affiliations:** 1 Department of Mechanical Engineering, University College London, Torrington Place, London WC1E 7JE, UK; 2 Department of Physics and Astronomy and London Centre for Nanotechnology, University College London, London WC1E 6BT, UK; 3 Maidstone Hospital, Hermitage Lane, Maidstone ME16 9QQ, UK; 4 Department of Pharmaceutics, University College London School of Pharmacy, Brunswick Square, London WC1N 1AX, UK

**Keywords:** electrospraying, site-specific drug delivery, delayed release, Eudragit, prednisolone

## Abstract

Single and coaxial electrospraying was used to prepare Eudragit L100-55 polymer microparticles containing prednisolone as the active pharmaceutical ingredient. Different compositions of prednisolone and Eudragit L100-55 were used to develop five different formulations with different polymer : drug ratios. The resultant microparticles had a toroidal shape with a narrow size distribution. Prednisolone was present in an amorphous physical state, as confirmed by X-ray diffraction analysis. Dissolution studies were carried out in order to investigate the feasibility of the proposed system for site-specific release of prednisolone. The release rates were interpreted in terms of diffusion-controlled release. It was shown that utilization of pH-responsive Eudragit L100-55 could minimize the release of prednisolone in the acidic conditions of the stomach, which was followed by rapid release as the pH of the release medium was adjusted to 6.8 after the first 2 h. This is especially desirable for the treatment of conditions including inflammatory bowel disease and colon cancer.

## 1. Introduction

An efficient drug delivery system should meet multiple functional requirements to serve as an ideal means of delivery of an active pharmaceutical ingredient (API). These include improved solubility and stability of the drug, reduced dosage and frequency of administration, reduced adverse side effects, minimum toxicity, and foremost the ability to deliver the required amount of drug to the target location over a long period of time. Change in the environmental pH is one of the most frequently applied stimuli for the development of triggers for drug release. The development of a pH-sensitive drug delivery system is mainly established by adopting polymers that bear weak acid (e.g. carboxylic acid) or base (e.g. primary and tertiary amines) groups with a p*K*_a_ that allows sharp changes in the ionization state at the desired pH. As stated by Rodríguez *et al*. [1], an increase in the degree of ionization enables dramatic change in the conformation and also the affinity of the chains, both for the solvent and for the intra-chain interactions, causing either disassembly of the components or swelling–shrinkage of the covalent network. The sensitivity to change in pH can be tuned by changing the nature of the co-monomers that are used to synthesize the polymer.

Eudragit L100-55, a copolymer of methacrylic acid and ethyl acrylate, was used in this study. Eudragit L100-55 dissolves when the pH is 5.5 [2]. Hence, it is resistant to gastric fluid and therefore it can bypass the stomach and release the incorporated API into the small intestine. Eudragit polymers have been extensively used for development of oral dosage formulations including tablet coatings and tablet matrices [3]. In addition to that, these polymers have also been used for preparation of microspheres and nanoparticles for controlled drug release systems in the gastrointestinal (GI) tract [4]. In a study by Hao *et al*. [5], Eudragit L100-55 was used as an enteric coating agent for encapsulation of aspirin in which the drug stability in the acidic environment of the upper GI tract was preserved while achieving mild cytotoxicity *in vitro*. A novel method to develop pH-responsive microparticles was reported by Alhnan *et al*. [6] in which they prepared formulations from aqueous solutions through spray-drying. They used Eudragit S and Eudragit L in order to develop gastric-resistant microparticles and successfully achieved fast and localized drug release at the small intestine. Hao *et al*. [7] used Eudragit L100-55 to develop enteric nanoparticles incorporating omeprazole, which is unstable in an acidic environment. Here, the emulsion diffusion method in conjunction with ultrasonic solidification was used to prepare the pH-sensitive drug delivery systems. In another study by Huanbutta *et al*. [8] electrohydrodynamic atomization was used to prepare prednisolone-loaded microparticles using Eudragit S. High encapsulation efficiency and compatibility of the proposed system was highlighted by these authors nonetheless no release study was conducted in order to further examine the proposed drug delivery system.

Colon-specific drug delivery is particularly advantageous for treatment of inflammatory bowel disease (IBD), which includes ulcerative colitis and Crohn's disease. Furthermore, oral delivery has been proven to be the most convenient route of administration of drug to patients [9]. Nonetheless, the variation in pH that a drug is exposed to, before it reaches the desired release location, remains an obstacle. Localized delivery of drugs is a prerequisite for optimum treatment of these chronic conditions, which require sustainable treatment. Thus, development of targeted delivery of an API to a specific region of the colon, where the API is protected from premature absorption in the upper GI tract, is of great significance. The variation in pH along the GI tract has been exploited for this purpose. In a study by Kendall *et al*. [10], the oil-in-oil emulsion technique was used for fabrication of pH-responsive acrylic microparticles for targeted GI delivery of prednisolone using Eudragit L55, S and L. It was discovered that Eudragit L was the most fitting polymer for this purpose, because it displays minimal drug release under acidic conditions and achieves optimum drug release when it reaches the pH threshold of the corresponding polymer carrier.

Prednisolone is among the most frequently prescribed drug for treatment of IBD. It is a corticosteroid that is widely used as both an immunosuppressant and an anti-inflammatory drug to treat a number of inflammatory and autoimmune conditions. However, because high dosages are used at frequent predetermined time intervals, it also induces adverse side effects [11,12]. Furthermore, it has poor water solubility, a characteristic of class II substances according to the Biopharmaceutics Classification System [13]. This is one of the major challenges in the development of oral formulations and is proven to be a limiting factor in drug bioavailability, which is directly related to the dissolution rate of the drug. Accordingly, various techniques including solid dispersion and preparation of liquisolid compacts [14,15] have been used for enhancement of the water solubility of prednisolone. Nonetheless, recent reports show that prednisolone-loaded particles have a low encapsulation efficiency [16]. In a study by Bílková *et al*. [17], prednisolone drug delivery systems with a highly selective release profile were developed using α-amino-ω-methoxypoly(ethylene glycol) and star α-aminopoly(ethylene glycol). They studied different approaches for obtaining rate-controlled release profiles and reported successful development of a drug delivery system that offers the potential for targeted release of prednisolone in transplanted liver. In another study by Bílková *et al*. [18], a drug delivery system with a delayed release profile of prednisolone was implemented, based on transient molecular protection of the pH-sensitive linker with α-cyclodextrin at the molecular level. The prepared polypseudorotaxane offered a promising oral delivery system with a pH-sensitive linker which exhibited delayed acid-catalysed hydrolysis.

Electrospraying has been investigated thoroughly as a potential technique for pharmaceutical applications [19] and more specifically for dissolution enhancement of water-insoluble drugs by facilitating preparation of the drug delivery system at reduced size [20], where a higher surface area to volume ratio greatly favours the rate at which the incorporated drug is released [21,22]. In a study by Zhang *et al*. [23], it was reported that using coaxial electrospraying for preparation of a griseofulvin formulation using Eudragit L100 as an encapsulating carrier significantly enhanced the dissolution and absorption behaviour of the poorly water-soluble drug. This was deemed to be due to the noticeable reduction in size of the drug carrier, enhanced dispersity and complete amorphization of the drug. A novel drug delivery system was developed by Wang *et al*. [24], who used a single nozzle emulsion electrospraying technique for co-encapsulation of multiple model drugs. They demonstrated that, by taking control of the regional drug loading, the release pattern could be modulated via careful positioning of incorporated active ingredients and appropriate selection of the polymer carrier. Alhnan *et al*. [25] carried out a study to examine the effects of microparticle and drug properties, including the size of the carrier and the drug molecular weight as well as the acid solubility of the drug, on undesired drug release in an acidic medium from enteric microparticles. They examined nine different drugs with variable chemical properties in combination with Eudragit L and S, and concluded that the molecular weight of the drug is the most crucial determinant for optimum limitation of drug release in an acidic environment.

In this work, electrospraying was exploited for development and characterization of prednisolone-loaded microparticles to prepare oral formulations for site-specific delivery using Eudragit L100-55 as the pH-responsive polymeric carrier. Five different formulations were prepared using single and coaxial electrospraying set-ups, with varying polymer : drug ratios, with the aim of minimizing the drug release in the acidic environment and achieving rapid release when the pH is adjusted to the pH threshold of the polymeric carrier. The solid state and release profiles of the developed formulations were studied and quantified.

## 2. Material and methods

### 2.1. Materials

Prednisolone crystalline powder was acquired from Sigma-Aldrich (Poole, UK) (MW = 360.44 g mol^−1^). Eudragit L100-55 (MW = 320 000 g mol^−1^) was supplied by Evonik GmbH (Germany). All other chemicals in this work were of analytical grade and used as provided.

### 2.2. Preparation and characterization of solutions

**Figure 1.**
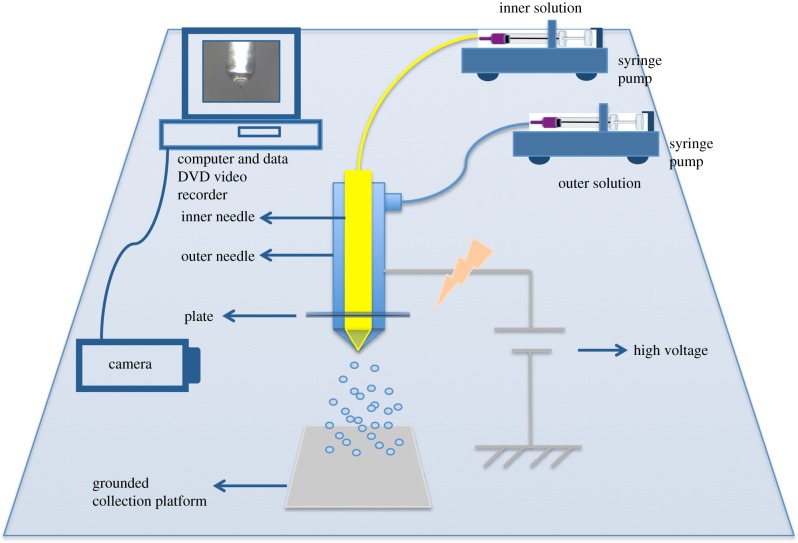
Schematic diagram of the coaxial electrohydrodynamic atomization technique. (Online version in colour.)

Spraying solutions were prepared by dissolving Eudragit L100-55 and prednisolone in isopropanol and methanol, respectively, and stirring using a magnetic stirrer to ensure formation of homogeneous solutions. All solutions were made under ambient conditions (room temperature 21 ± 1°C and relative humidity of 40–60%). Eudragit solutions of 1.0% w/w and 2.0% w/w were prepared by dissolving the polymer in isopropanol. Prednisolone solutions of 0.1% w/w and 0.2% w/w were prepared by dissolving the appropriate amount of drug in methanol. Solutions of prednisolone 0.2% w/w and Eudragit L100-55 1.0% w/w were prepared by dissolving the appropriate amount of drug and polymer in methanol. Prednisolone 0.1% w/w and Eudragit L100-55 1.0% w/w were dissolved in isopropanol. The composition details of the prepared solutions are shown in table 1, where they are denoted as F1–F5. Solutions were fed onto 10 ml airtight syringes to avoid formation of bubbles during processing. Concentric needles of inner diameter 0.6 mm and 1.52 mm and outer diameter of 0.9 mm and 2.03 mm, respectively, were connected to a high-precision voltage power supply (figure 1).

**Table 1.** Formulations and compositions of F1–F5.

formulation	core solution	shell solution
F1	prednisolone 0.1% w/w in MeOH	L100-55 2% w/w in isopropanol
F2	prednisolone 0.2% w/w in MeOH	L100-55 1% w/w in isopropanol
F3	prednisolone 0.2% w/w in MeOH	L100-55 2% w/w in isopropanol
F4	prednisolone 0.2% w/w and L100-55 1% w/w in MeOH	L100-55 2% w/w in isopropanol
F5	single - prednisolone 0.1% w/w and L100-55 1% w/w in isopropanol	none

The positive electrode of the high-precision DC voltage power supply (HCP 35-65000; Fug Elektronik, Rosenheim, Germany) was connected to a metal needle tip. The ground electrode was connected to a metal collector plate. Experiments were carried out under ambient conditions. Electrical potential was applied throughout a fixed distance of 250 mm between the concentric needle tip and the collection platform. The flow rate was kept constant for formulations F1, F2, F3, F4 at 6 µl min^−1^ and 12 µl min^−1^ for the inner and outer solution, respectively. The flow rate for F5 was set at 12 µl min^−1^. The applied voltage was varied from 14 kV to 19 kV, in order to establish a stable cone jet. Different formulations were prepared using single and coaxial needle configurations, in order to examine the release profile of prednisolone combined with different polymer concentrations.

The characteristic properties of the prepared solutions, including electrical conductivity, surface tension, viscosity and density, were measured under ambient conditions (table 2). Electrical conductivity was determined using a conductivity probe (Jenway 3540 pH/conductivity meter). Viscosity was measured using a U-tube viscometer (75 ml Cannon-Fenske Routine Viscometer; Cannon Instruments, USA), and a Kruss tensiometer (model-K9; Kruss GmbH, Germany) was used to determine the surface tension. The viscometer, tensiometer and electrical conductivity probe were calibrated before measurement. Methanol has a higher electrical conductivity than that of isopropanol. Both solvents had nearly the same surface tension with measured values of 27.0 ± 0.2 mN m^−1^ and 26.7 ± 0.2 mN m^−1^ for methanol and isopropanol, respectively. Isopropanol is slightly more viscous than methanol with measured viscosities of 1.4 ± 0.1 mPa s and 0.7 ± 0.2 mPa s, respectively.

**Table 2.** Properties of the prepared solutions.

solution	surface tension (mN m^−1^)	viscosity (mPa s)	electrical conductivity (μS cm^−1^)
prednisolone 0.1% w/w in MeOH	23.4 ± 0.2	0.8 ± 0.3	10.0 ± 0.1
prednisolone 0.2% w/w in MeOH	24.4 ± 0.3	0.8 ± 0.3	2.5 ± 0.1
prednisolone 0.1% w/w + L100-55 1% w/w in isopropanol	25.2 ± 0.5	2.5 ± 0.5	3.2 ± 0.1
prednisolone 0.2% w/w + L100-55 1% w/w in MeOH	25.5 ± 0.4	0.9 ± 0.3	30.6 ± 0.4
L100-55 2% w/w in isopropanol	26.2 ± 0.3	2.8 ± 0.1	5.3 ± 0.1
L100-55 1% w/w in isopropanol	25.8 ± 0.2	2 ± 0.1	3.2 ± 0.1

### 2.3. Characterization of particle size and morphology

#### 2.3.1. Scanning electron microscopy

The morphology of the developed formulations was assessed using a scanning electron microscope (SEM; XL30 FEG; Philips). Using ImageJ software, particle size distribution, average diameter and standard deviation of the particle population were assessed by measuring the size of 300 particles for each developed formulation.

#### 2.3.2. X-ray diffraction

X-ray diffraction (XRD) patterns were acquired using a MiniFlex600 diffractometer (Rigaku, Tokyo, Japan), with Cu Kα radiation (*λ* = 1.5418 Å) at 40 kV and 15 mA. Data were obtained over the 2*θ* range 5–45° and at a scan speed of 2° min^−1^.

#### 2.3.3. Differential scanning calorimetry

Differential scanning calorimetry (DSC) measurements were performed using a TA Instruments Q1000 Differential Scanning Calorimeter in order to assess the physical state of the drug within the formulations. Samples of pure drug and polymer and the various formulations were weighed (3–4 mg) in pierced aluminium pans and evaluated over the temperature range of 40–270°C at a heating rate of 10°C min^−1^.

#### 2.3.4. Fourier transform infrared spectroscopy

A Spectrum 100 spectrometer (PerkinElmer, Massachusetts, USA) was used to record Fourier transform infrared (FTIR) spectra. The spectrometer was fitted with an attenuated total reflectance attachment (ATR-FTIR). The samples were scanned over the range of 4000–650 cm^−1^, with the resolution set at 1 cm^−1^.

#### 2.3.5. Drug encapsulation efficiency

A total of 20 mg of each formulation was dissolved in 20 ml ethanol to determine the actual amount of drug encapsulated in the particles with respect to its theoretical value. This was done in order to measure the encapsulation efficiency of each formulation. The following equation was used for the calculations:2.1
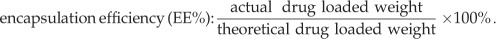


#### 2.3.6. *In vitro* drug release

A total of 50 mg of particles was placed in an empty capsule. The capsules were loaded onto enclosed metallic sinkers. The sinkers were attached to the rotating rods in the dissolution apparatus. The beakers were filled with buffer solution under 50 r.p.m. continuous stirring at a set temperature of 37 ± 0.5°C. *In vitro* drug dissolution tests were conducted in 750 ml hydrochloric acid solution pH 1.2 for the first 2 h. Thereafter, the pH of the dissolution medium was adjusted to 6.8 for the following 6 h. The release studies were conducted according to the United States Pharmacopeia standards. At predetermined periodic intervals, 3 ml aliquots were withdrawn from the dissolution medium and replaced with 3 ml of preheated fresh buffer solution to ensure a constant volume and maintain sink conditions. The drug concentrations in the withdrawn aliquots were obtained using UV spectrometry (6305 spectrophotometer; Jenway, UK). The detection wavelength was set at 247 nm. Experiments were conducted in triplicate and results recorded as mean ± s.d.

## 3. Results and discussion

### 3.1. Morphology

The solution fed into the process, in electrospraying, undergoes various modes. The establishment of different modes exhibited is interplay between solution physico-chemical characteristics and experimental parameters. Experimental parameters, namely the applied voltage, the set flow rates and the working distance between the needle exit and the collection platform, are the primary parameters that determine the window at which a stable cone jet can be formed. In addition to the operating parameters, the properties of the liquid, including the electrical conductivity and viscosity, govern the establishment of the stable cone jet mode [26]. For all formulations, any applied voltage lower or higher than that of a stipulated range resulted in the formation of an unstable jet or multiple jets, which would in turn result in the formation of particles with a large size distribution. The consistent working distance and set flow rates were chosen to minimize significant size variation among developed formulations. This enabled limitation of the potential factors affecting this variance, which is deemed to be favourable in terms of enabling correct analysis of the release profiles obtained from each formulation and isolating the primary factors leading to achievement of the different release patterns.

**Figure 2.**
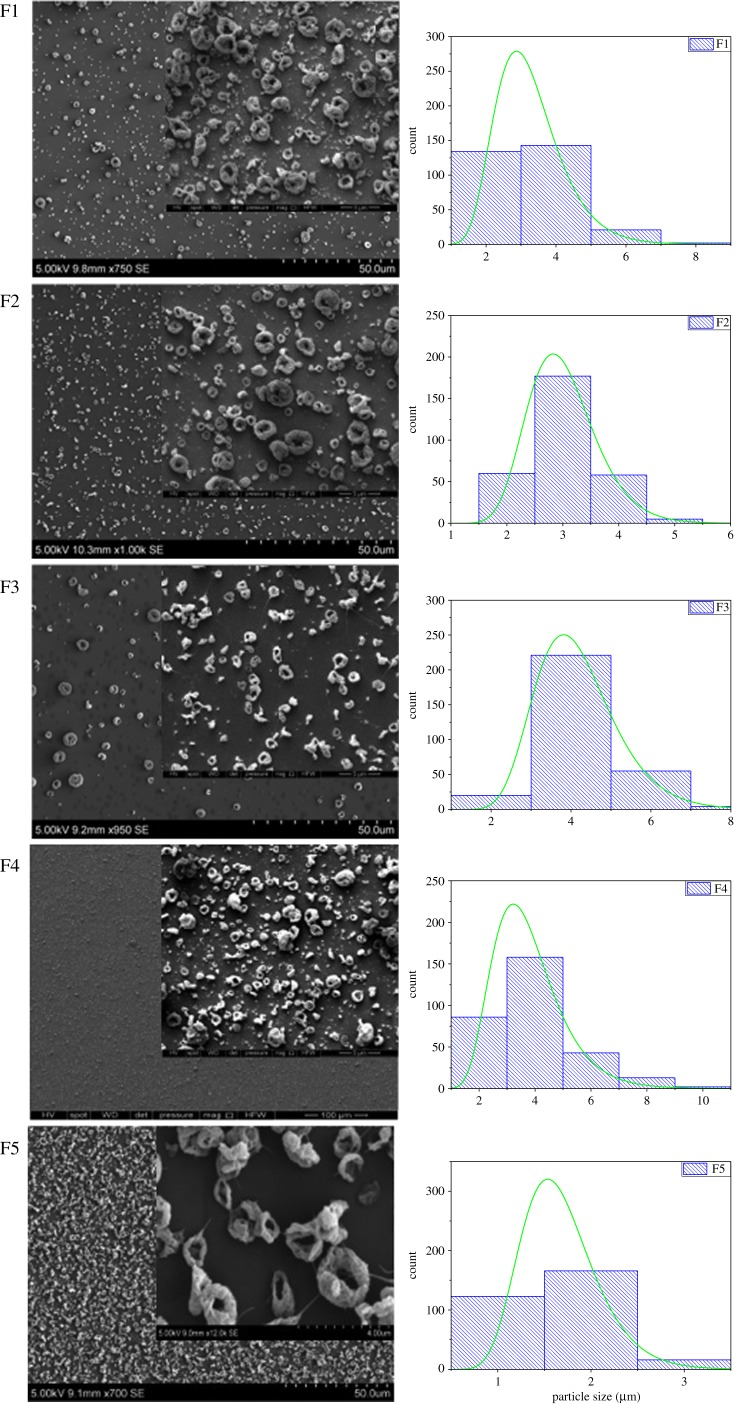
Size distribution graphs and scanning electron microscopy images of the particles prepared from formulations F1–F5. (Online version in colour.)

The particle morphology of the prepared formulations is demonstrated in figure 2; as can be deduced from the SEM images, the particles adopted the form of doughnut-shaped encapsulating matrices. The hypothesis is that the periphery of the droplet is pinned as the solvent is evaporating further [27]. Solvent removal from the surface results in an increase in the concentration of the polymer near the surface; this culminates in the formation of toroidal structures. Use of high-molecular-weight Eudragit L100-55 (320 kDa) resulted in limited mobility of the polymer solution as the drying process occurred. For this reason, the formation of spherical particles was prohibited. The formation of toroidal structures was consistent in all formulations, although the polymer concentration was kept at the lowest possible value due to the high viscosity of the prepared solution, while at the same time enabling production of particles.

In terms of size, F1 and F3 formulations consisted of the same outer solution, with F3 having a higher drug concentration in the core needle (table 1). The average applied voltage was slightly higher for F1, at 19 kV, than for F3, 16 kV. Hence, the F1 average particle size was 3.2 ± 1.0 µm as opposed to 4.2 ± 1.0 µm for F3, as shown in table 3; this is consistent with the literature [28]. The outer solution in F4 also has the same polymer concentration as F1 and F3, but, in this formulation, the inner solution consisted of both the drug and polymer, in order to further decrease the drug release in the first 2 h before it reaches the intestine. Inclusion of polymer, as well as having relatively lower applied voltage of 15–18 kV, resulted in a higher average particle size than that of F1, at 3.8 ± 1.4 µm (figure 2). Also, when compared with F3, F4 has a lower than average particle size, where the applied voltage is slightly lower for F3 at 16 kV. The constituent of the inner solution for F2 and F3 was kept unchanged; however, the outer solution in F3 had a higher polymer concentration than that of F2, and the average particle size was 3.0 ± 0.6 µm at an applied voltage of 18–19 kV for F2, and 4.2 ± 1.0 µm at an applied voltage of 16 kV for F3. The slightly higher particle size of F3 can be explained by the higher polymer content, as well as the lower applied voltage. Among all the developed formulations, F5, for which single needle electrospraying was used, had the lowest particle size at 1.7 ± 0.4 µm and applied voltage of 14–19 kV. Although the polymer : drug ratio for F5 was the same of that for F2, having no outer shell solutions resulted in a reduction in particle size.

**Table 3.** Encapsulation efficiency and mean particle size data for the prepared formulations.

formulation	mean particle outer diameter (μm)	mean particle inner diameter (μm)	encapsulation efficiency (%)
F1	3.2 ± 1.0	0.7 ± 0.2	93
F2	3.0 ± 0.6	0.5 ± 0.1	81
F3	4.2 ± 1.0	0.5 ± 0.2	83
F4	3.8 ± 1.4	0.4 ± 0.1	84
F5	1.7 ± 0.4	0.6 ± 0.2	76

### 3.2. Phases

**Figure 3.**
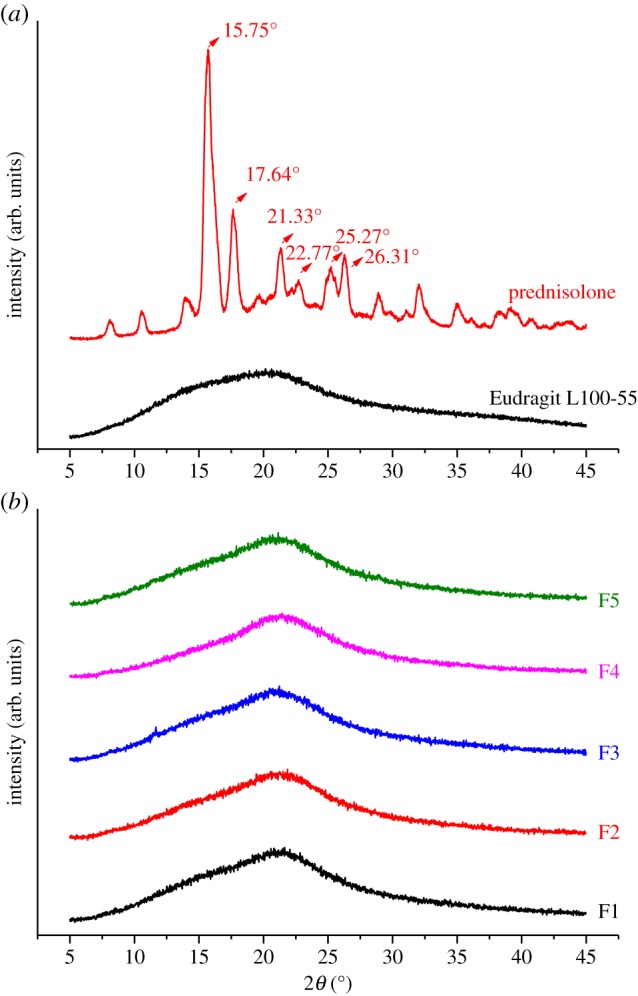
X-ray diffractograms of (*a*) prednisolone and Eudragit L100-55 and (*b*) the developed formulations (F1–F5). Arbitrary units are indicated by arb. units (Online version in colour.)

The XRD patterns of the polymer, Eudragit L100-55, and the drug, prednisolone, and also those of the developed formulations are illustrated in figure 3. Prednisolone exhibited numerous reflections and sharp peaks at 15.75°, 17.64°, 21.33°, 22.77°, 25.27° and 26.31°, demonstrating its crystallinity. Eudragit L100-55 displayed the characteristic broad hump of amorphous materials. As shown in the diffractograms obtained for the developed formulations, the amorphous nature was most prominent. In all formulations, the characteristic peak of prednisolone at 2*θ* of 15.7° was found to be invisible and overlapped with that of Eudragit L100-55 at 2*θ* of 20.76°. As illustrated by the superimposed X-ray diffractograms of the formulations, the amorphous state of prednisolone within the formulations can be established, confirming that the drug is molecularly dispersed in Eudragit L100-55.

### 3.3. Thermal properties

**Figure 4.**
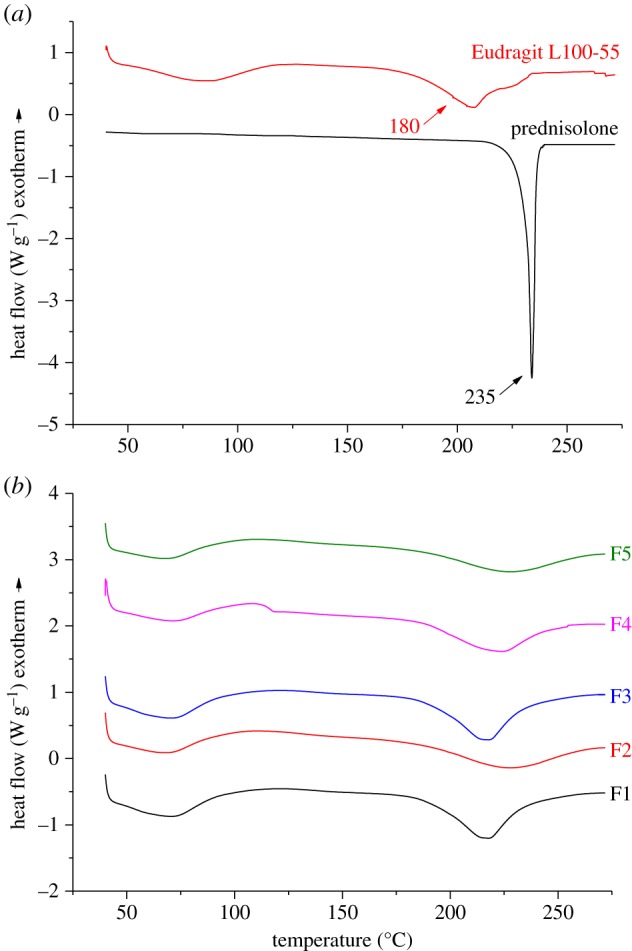
DSC thermograms of (*a*) prednisolone and Eudragit L100-55 and (*b*) the developed formulations (F1–F5). (Online version in colour.)

DSC analyses were performed in order to evaluate possible solid-state interactions between the components and, consequently, to assess the actual drug–excipient compatibility in all the examined formulations. The thermal curves of pure components, prednisolone and Eudragit L100-55, and those of the developed formulations (F1–F5) are shown in figure 4.

The DSC curve of the pure drug is indicative of its crystalline state, displaying a sharp endothermic peak at 235°C. The thermal profile of Eudragit L100-55 exhibited a broad endothermic band that ranged between 50°C and 100°C, due to dehydration as evaporation of unbound water took place. The second endothermic effect was observed at a higher temperature, and is attributable to the melting of its crystalline phase. Eudragit L100-55 is a heat-labile polymer that undergoes thermal degradation at 150°C by decomposition of carboxylic side groups, followed by chain decomposition at 180°C [29]. The thermal curves of the developed formulations (F1, F2, F3 and F4) almost corresponded to those of the pure components, demonstrating the absence of solid-state interactions and allowing assessment of drug–polymer compatibility in all the examined formulations. As shown from the thermograms obtained for F1, F2, F3 and F4, the melting temperature of prednisolone shifted to a lower value as the L100-55 has acquired a higher degradation temperature, and the new *T*_m_ is at an intermediate point between the two. This shift is steepest for F1, where the polymer : drug ratio is at its highest; this may be due to random dispersion of prednisolone molecules within the polymeric carrier and a semi-crystalline structure.

### 3.4. Compositional features

**Figure 5.**
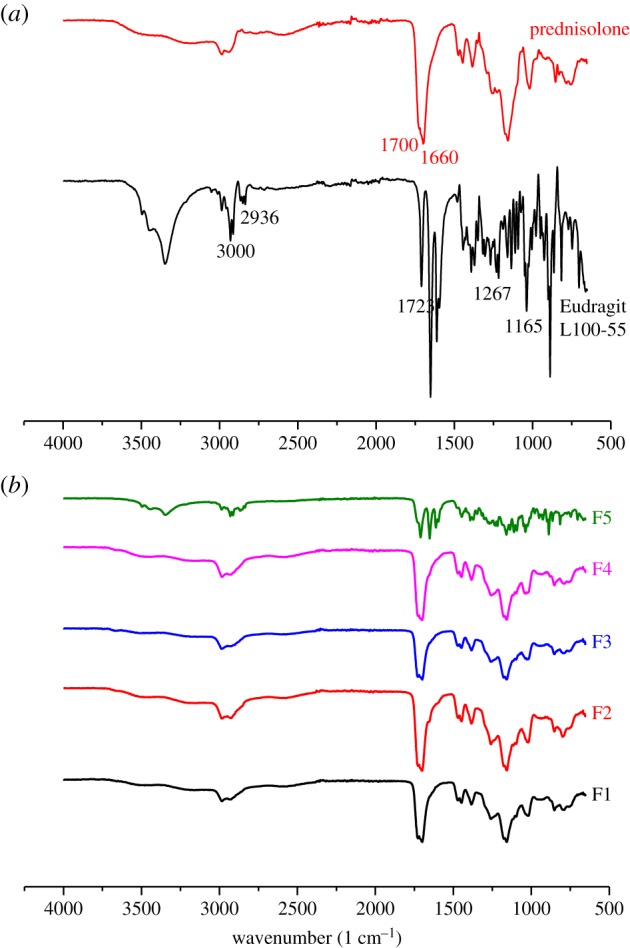
FTIR spectra of (*a*) prednisolone and Eudragit L100-55 and (*b*) the developed formulations (F1–F5). (Online version in colour.)

FTIR spectroscopy was used in order to characterize possible interactions between the drug and the polymer in the solid state. FTIR spectra of the unprocessed material, the unloaded and loaded formulations and the physical mixture were obtained as illustrated in figure 5. The spectrum of prednisolone showed characteristic bands of the OH group at 3200–3500 cm^−1^ (OH involved in intermolecular association). The characteristic stretching vibrations of C=O at 1700 cm^−1^ and C=C at 1660 cm^−1^ appear as very strong bands. The spectrum of Eudragit L100-55 shows characteristic bands of methyl and methylene CH stretch vibrations at 3000 cm^−1^ and 2936 cm^−1^, a strong band owing to carbonyl groups at 1723 cm^−1^ (C=O stretch) and two bands at 1267 cm^−1^ and 1165 cm^−1^ that are due to ester linkages (COC stretches).

### 3.5. Drug release

**Figure 6.**
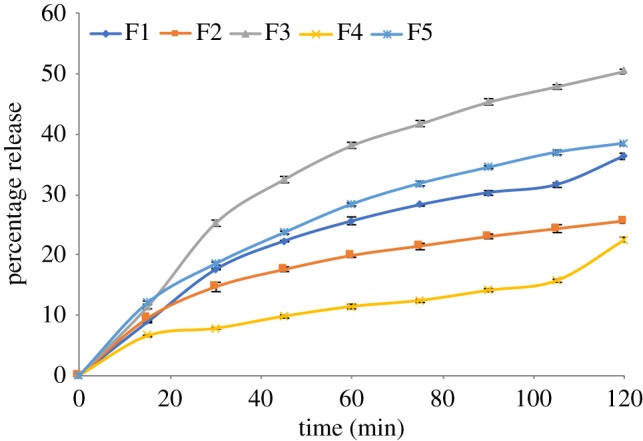
Percentage drug release for the first 2 h (F1–F5).

**Figure 7.**
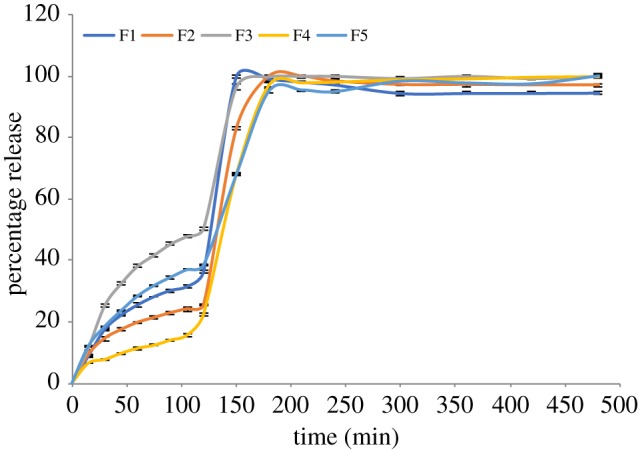
Percentage drug release (F1–F5).

To investigate prednisolone release profiles from Eudragit L100-55 developed formulations, *in vitro* dissolution tests were conducted on F1–F5 under acidic conditions pH 1.2 for the first 2 h, which replicates the stomach passage transit time, followed by 6 h at pH 6.8, which mimics intestinal conditions. The drug release profiles are shown in figures 6 and 7. The encapsulation efficiencies obtained for all formulations are presented in table 3.

Prednisolone release from all formulations is retarded to different extents during the initial 2 h of release in an acidic environment, depending on the composition of the formulation. This is due to the insolubility of Eudragit L100-55 at pH < 5.5. It can be seen from the release graphs that the prednisolone release during the first 2 h is at its highest for F3 > F5 > F1 > F2 > F4. Although the polymer : drug ratio is kept the same for F2 and F5, the percentage release for the first 2 h is higher for F5, where the polymer and drug were fed into single needle electrospraying, as opposed to F2, where the drug solution was fed only into the inner needle. Moreover, the mean particle size corresponding to F5 is lower at 1.7 ± 0.4 µm than that corresponding to F2 with a mean particle size of 3.0 ± 0.6 µm. It was also found that the drug encapsulation efficiency was higher for F2 at 81% than for F5 at 76%. The drug release was at its least for F4, whereby Eudragit L100-55 was incorporated in both the inner and outer solutions fed into the coaxial electrospraying, where prednisolone was only in the core solution. The encapsulation efficiency was at peak value for F1, 93%, where the polymer concentration with respect to drug was at its highest.

After the first 2 h, when the pH is at 6.8, F1 shows the fastest initial dissolution rate. Prednisolone release exhibits a more sustained pattern for F4 and F5 when the drug release reaches a plateau after 3 h. The drug release rate in μg ml^−1^ follows the order F2 > F5 > F3 > F1 > F4. Total drug release over a longer time span is shown in figure 7. Although Eudragit L100-55 is soluble at pH > 5.5, the drug dissolution process did not occur instantly, which shows that further physical processes take place before the incorporated drug reaches its maximum release amount in the release medium. Particles need to absorb water, swell and disentangle prior to complete dissolution of the incorporated drug. The obtained results show that both the polymer : drug concentration ratio and solution compositions using single and coaxial electrospraying set-ups can affect both encapsulation efficiency and dissolution rate. It can be concluded that using polymer in both the core solution and the shell solution can further prolong the release and yield higher encapsulation efficiency.

### 3.6. Modelling the drug release

The micrographs of the particles suggest that the morphology is more toroidal than spherical, so we have considered the effect of this on the release rates. Unfortunately, the time-dependent diffusion equation is not separable in toroidal coordinates. It is possible [30] to establish a hierarchy of coupled partial differential equations representing an expansion in powers of *r*/*R*, where *R* is the radius of the toroid and *r* is the radius of the tube which forms the toroid. The first term in the expansion is the same as the result for an infinite cylinder of radius *r*, and the lowest-order correction is proportional to (*r*/*R*). To investigate further we have undertaken numerical calculations of the release rates from toroids using a finite-element method in the FreeFEM++ code [31]. We consider particles in which an active ingredient is initially uniformly distributed, and diffuses with coefficient *D* into a surrounding material in which the concentration is zero. We found that the rate of release is independent of the ratio *r*/*R*, and is numerically identical to the rate of release from a cylinder of radius *r*. Accordingly, we have fitted the early stage release rates by using the following expression, which we have found to give a good fit to well-known exact analytical results [32]:3.1
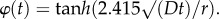


For comparison, we have also used the expression previously derived [33] for spheres of radius *a*,3.2
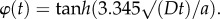


We note that both expressions can be represented by the functional form tan*h*(*α*√*t*): the interpretation of *α* in terms of a diffusion coefficient depends on the particle size and shape.

The release rate in a less acidic environment is so rapid that the experimental measurements are not closely enough spaced to justify detailed numerical fits: nevertheless, we have again used the expressions in equations (3.1) and (3.2) to describe the release after 2 h in order to gain an order-of-magnitude estimate of the change in diffusion constant in Eudragit in the two environments. We do not attempt to make any allowance for dimensional changes of the particles.

**Figure 8.**
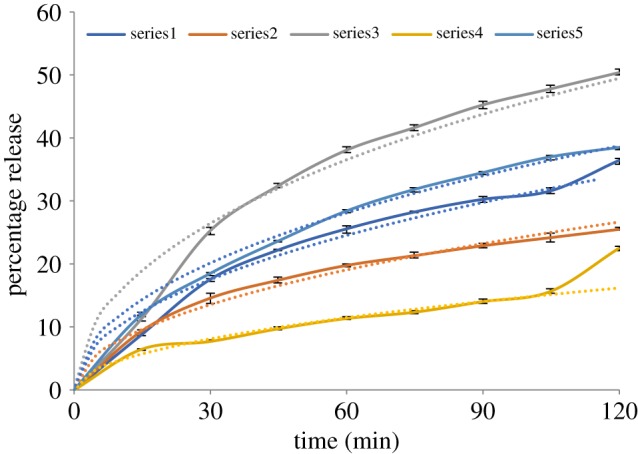
Observed (solid) and fitted (dashed) release profiles for the five particle formulations (F1–F5) during the first 2 h.

Figure 8 compares the best fits to the release rates for the first 2 h with the experimental results. We note that the fits are rather poor over the first minutes of the release, suggesting that a slightly different mechanism is in operation during these very early stages. If these fits are interpreted in terms of a diffusion coefficient, the results are as shown in table 4. It is notable that the dependence of the results on the assumed geometry is weaker than the dependence on the particle formulation. The release rate curves clearly show an increase in rate after the decrease in pH of the surrounding solution.

**Table 4.** Diffusion coefficients *D* for the first 2 h of release deduced from fitting the experimental results with equations (3.2) and (3.1).

formulation	sphere diameter (μm)	*D*/(m^2^ s^−1^) (sphere)	cylinder diameter (μm)	*D*/(m^2^ s^−1^) (cylinder)
F1	3.2	4.0 × 10^−18^	1.25	4.7 × 10^−18^
F2	3.0	2.1 × 10^−18^	1.25	2.8 × 10^−18^
F3	4.2	1.6 × 10^−17^	1.85	2.4 × 10^−17^
F4	3.8	1.2 × 10^−18^	1.70	1.8 × 10^−18^
F5	1.7	1.5 × 10^−18^	0.55	1.2 × 10^−18^

### 3.7. Clinical perspective

IBD comprises predominantly ulcerative colitis and Crohn's disease—two complex, chronic conditions. Crohn's disease is characterized by transmural inflammation affecting anywhere along the GI tract, most commonly the terminal ileum. Ulcerative colitis is described as mucosal inflammation restricted to the colon, often affecting the distal colon in a continuous manner. Symptoms can include severe diarrhoea and abdominal pain, frequently presenting with weight loss and malaise. Both conditions have the capacity to greatly affect an individual's day-to-day life and, in some cases, pose life-threatening emergencies. The variable nature of IBD in each patient means we are faced with challenges in providing effective treatment. The anti-inflammatory effects of corticosteroids such as prednisolone can induce remission and prevent acute flares of the disease.

Targeted drug delivery to the bowel is highly desirable owing to the changing environment along the GI tract. The steroids taken via the oral route are susceptible to changes in pH, enzymatic action and prolonged transit times, compromising absorption. Our understanding of the gut physiology can however be used to our advantage. To tailor the drug distribution to the section of bowel affected, one must understand the changes in pH along the GI tract. The stomach tends to be extremely acidic, the pH then becomes more alkaline moving along the small intestine, reaching approximately 7.4 at the distal small intestine. Moving into the colon, the pH drops at the caecum to approximately 5.7, rising once again to 6.8 at the rectum. The pH can further be influenced by dietary intake, resident gut flora and disease-induced inflammation. Having a prednisolone coating that is initially resilient to acidic pH but then highly responsive to a higher pH allows for a much more efficient and successful delivery. Tailoring experiments to exact pH variation is however difficult, but the work described in this paper furthers this aim significantly.

Prolonged use of steroids unfortunately comes with systemic side effects including hyperglycaemia, gastric irritation and osteoporosis. Formulations which are pH responsive are therefore highly attractive to both the prescriber and the patient. By optimizing where the drug acts we can reduce the dose and frequency required, restricting the undesired effects. Through optimizing the safety and efficacy of prednisolone, we have the potential to greatly improve patient compliance and reduce costs of administering excessive quantities of steroids. Prednisolone is implicated in the treatment of many bowel pathologies, including carcinoma of the bowel, the prevalence of which is rising rapidly. The carefully constructed drug formulations described are able to work in combination with our gut physiology rather than against it, which makes a wide range of future applications possible.

## 4. Conclusions

We report successful preparation of a series of five formulations of prednisolone for localized delivery using electrospraying. These include toroidal microparticles composed of pH-responsive Eudragit L100-55. XRD showed that prednisolone was in an amorphous state in the formulations. Dissolution studies showed that the proposed drug delivery system was successful in terms of minimization of prednisolone release within the first 2 h under the acidic conditions of the stomach at pH 1.2, this was followed by immediate release after the adjustment of the pH to 6.8. The slower release in the early stages could be described by a diffusion process. The developed formulations could offer localized delivery of prednisolone to the colon, where site-specific release of the API is anticipated for treatment of inflammatory bowel syndrome or colon cancer. It was proved that electrospraying offers a versatile platform for preparation of targeted release formulations by careful selection of the polymeric system composition that is highly adaptable to obtain different modes of release based on the desirable requirements.

## Data accessibility

Data supporting this study are provided in the paper.
